# The role of the immune system and the biomarker CD3 + CD4 + CD45RA−CD62L− in the pathophysiology of migraine

**DOI:** 10.1038/s41598-020-69285-4

**Published:** 2020-07-23

**Authors:** Zbyšek Pavelek, Ondřej Souček, Jan Krejsek, Lukáš Sobíšek, Blanka Klímová, Jiří Masopust, Kamil Kuča, Martin Vališ

**Affiliations:** 1 Department of Neurology, Faculty of Medicine, University Hospital Hradec Králové, Charles University, Sokolská 581, Prague, Hradec Králové, 500 05 Czech Republic; 2 Department of Clinical Immunology and Allergology, University Hospital Hradec Králové, Hradec Králové, Czech Republic; 3 Biomedical Research Center, University Hospital Hradec Kralové, Hradec Kralové, Czech Republic; 4 Department of Chemistry, University of Hradec Kralove, Hradec Kralove, Czech Republic

**Keywords:** Neuroscience, Neurology

## Abstract

The role of the immune system as an integral component of the inflammatory response in the pathophysiology of migraine remains unclear. The aim of this study was to evaluate the differences in immune system parameters (acquired immunity parameters) in patients with episodic migraine (EM) and in healthy controls. In EM patients, we aimed to determine whether the changes found in peripheral blood parameters were related to migraine severity according to the standardised MIDAS and HIT-6 tests. Forty-nine patients with EM and 50 healthy controls were included in this study. The authors compared different lymphocyte parameters obtained by multicolor flow cytometry in the EM and control groups by performing statistical tests. The relationship between the changes in peripheral blood parameters and migraine severity in EM patients was investigated using correlation and regression analysis. EM patients showed higher values than healthy controls, especially in nine parameters: relative count of lymphocytes, relative and absolute counts of CD3 T cells, relative and absolute counts of CD8 suppressor cytotoxic T cells, relative and absolute counts of CD4 + T_EMRA_ (terminally differentiated helper T lymphocytes), absolute count of CD8 naïve T cells, and absolute count of CD19 switched memory B cells. Among the lymphocyte parameters, CD4 + T_EM_ (effector memory helper T lymphocytes) and CD8 + T_EMRA_ (terminally differentiated cytotoxic T lymphocytes) were statistically significantly associated with HIT-6. Patients with a CD4 + T_EM_ value below 15 had a high probability (90%) that the HIT-6 value would be higher than 60. The results of this study show that EM patients have changes in immune system parameters measured in the peripheral blood. Changes in the abundance of CD4 + T_EM_ could be used as a biomarker for disease severity.

## Introduction

Migraine is the sixth most common cause of disability worldwide, and it significantly worsens the quality of life of affected individuals and places a great economic burden on society^1^. An extensive European research study reported a big effect of migraine among 17.7% of men and 28.0% of women losing within 10 days of activities during a 3-month period^2^. Sokolovic et al.^3^ revealed that persons with headaches lost 10.2 workday equivalents per year (estimated from absenteeism and days with productivity reduction ≥ 50%, typically without including days with productivity reduction < 50%)^3^. An American study stated that, of the total $13 billion economic burden from migraine, impaired work function accounted for $5 billion and that direct medical costs only reached $1 billion^4^.

Intensive research in recent years has greatly increased the understanding of the pathophysiology of this disease and has contributed to the discovery of new drugs that were developed based on the knowledge of pathophysiological processes. A migraine headache is caused by the depolarisation of nociceptive trigeminal nerve fibres belonging to the trigeminovascular system. When the trigeminovascular system is stimulated, the vasoactive neurotransmitters are released from the perivascular nerve endings such as Calcitonin Gene-Related Peptide (CGRP), substance P and neurokinin A, all which cause vasodilation of the meningeal vessels and sterile perivascular neurogenic inflammation^5^.

The role of the immune system in the pathophysiology of migraine remains unclear^6^. Moskovitz first suggested the idea that local neurogenic inflammation due to the release of neuropeptides from meningeal nerves is involved in migraine^7^. Neurogenic inflammation occurs when vasoactive neurotransmitters, especially CGRP, release inflammatory mediators such as serotonin, histamine and prostaglandins from activated and degranulated dural mast cells. Anti-migraine drugs like sumatriptan or naratriptan can inhibit plasma protein extravasation in the dura mater^8,9^. On top of that, the efficacy of nonsteroidal anti-inflammatory drugs in the inhibition of dural plasma protein extravasation are additional arguments for a pathogenic role of meningeal neurogenic inflammation in migraine as well as the elevated CGRP levels that were found in plasma samples obtained from the jugular vein during a migraine attack^10^.

The results of a number of already published studies indicate altered immune function in migraineurs. Fidan et al. studied the role played by cytokines in migraine. They described significantly higher IL-6 levels in migraine patients^11^. Other studies from the last decade have shown, among other things, a reduction in CD4 + CD25 + regulatory T cells in migraine; changes in lymphocyte subsets in paediatric migraine (lower CD8 + prevalence and a higher CD4 + /CD8 + ratio in the ictal phase irrespective of migraine subtype); higher CD3, CD4, CD8 and CD19 in patients with chronic migraine compared to patients with episodic migraine; and an increased proportion of Treg CD45R0 + CD62L– and CD45R0–CD62L– cells^12–15^.

The purpose of this article was to evaluate the differences in immune system parameters (adaptive immunity parameters) in peripheral blood in patients with episodic migraine (EM) and in healthy controls. We also aimed to determine whether the changes found in peripheral blood parameters in EM patients were related to migraine severity according to the standardised MIDAS and HIT-6 tests and to thus find a biomarker that determines the severity of migraine.

## Materials and methods

### Study population

This observational study was conducted from January 2019 to May 2019. A total of 99 volunteers were enrolled in the study. Forty-nine volunteers were patients with episodic migraine. Fifty patients were healthy controls (HCs) without any comorbidities who had never experienced headaches in their life and were 18–65 years of age. All of the volunteers with EM were all patients of the Department of Neurology of the University Hospital in Hradec Králové, Czech Republic. The eligibility criteria for patients with EM included being aged 18–65 years with EM without aura based on the *International Classification of Headache Disorders* 3rd edition for migraine without aura^16^, a history of migraine without aura of at least 1 year, migraine onset prior to age 50, at least 1 migraine attack per month on average within the past 3 months and no comorbidities. During the analysis, no EM patients had used any prophylactic medication to date. The analysis (peripheral blood samples) was performed at least one week after the last episode of migraine.

All participants provided written informed consent. This study was approved by the ethics committee of the University Hospital Hradec Králové. The trial was registered under reference number 201809S18P.

### Flow cytometry and haematology

The authors collected blood samples from the antecubital fossa vein into sample tubes pre-coated with EDTA anticoagulant. The blood count was performed with a Sysmex XN‐3,000. For the surface staining of lymphocytes, 50 µl of blood was added to tubes containing 5 µl of fluorochrome-labelled monoclonal antibodies. All the antibodies used and their source information and dilution factors are summarised in Supplementary Table 1.

Blood samples were incubated with antibodies for 15 min at room temperature in the dark. Then a lysis solution (OptiLyse C, Beckman Coulter) was added, and the mixture was incubated for another 10 min. Flow cytometric evaluation was conducted with a Navios 10 flow cytometer (Beckman Coulter). All the data were then assessed using Kaluza 2.1 Analysis Software (Beckman Coulter). A minimum of 30,000 events were obtained for each stain and were supplied in list mode. Multiple peripheral blood parameters were assessed as absolute and relative values. The gating strategies for the different lymphocyte subsets assessed were as follows: lymphocytes (low SSC/CD45 + +), T lymphocytes (CD3 +), B lymphocytes (CD19 +), natural killer (NK) cells (CD3-CD16 + and/or CD56 +), helper T lymphocytes (CD3 + CD4 +), cytotoxic T lymphocytes (CD3 + CD8 +), naïve helper T lymphocytes (CD3 + CD4 + CD45RA + CD62L +), terminally differentiated helper T lymphocytes—CD4 + T_EMRA_ (CD3 + CD4 + CD45RA + CD62L-), central memory helper T lymphocytes (CD3 + CD4 + CD45RA-CD62L +), effector memory helper T lymphocytes—CD4 + T_EM_ (CD3 + CD4 + CD45RA-CD62L-), naïve cytotoxic T lymphocytes (CD3 + CD8 + CD45RA + CD62L +), terminally differentiated cytotoxic T lymphocytes—CD8 + T_EMRA_ (CD3 + CD8 + CD45RA + CD62L-), central memory cytotoxic T lymphocytes (CD3 + CD8 + CD45RA-CD62L +), effector memory cytotoxic T lymphocytes (CD3 + CD8 + CD45RA-CD62L-), regulatory T lymphocytes (CD3 + CD4 + CD25 +  + CD127-), regulatory T lymphocytes CD45RA + (CD3 + CD4 + CD25 +  + CD127-, CD45RA +), activated T lymphocytes (CD3 + CD69 +), activated B lymphocytes (CD19 + CD69 +), activated NK cells (CD3-CD56 + and/or CD16 + CD69 +), CD57-positive T lymphocytes (CD3 + CD57 +), CD57-positive NK cells (CD3-CD56 + and/or CD16 + CD57 +), naïve B lymphocytes (CD19 + IgD + CD27), non-switched memory B lymphocytes (CD19 + IgD + CD27 +), class-switched memory B lymphocytes (CD19 + IgD-CD27 +) and double-negative B lymphocytes (CD19 + IgD-CD27-). The gating strategy for the different lymphocyte subsets is presented in supplementary Figs. 1–3. The absolute values were calculated from the blood counts, and the relative values were calculated as the percentage of the population described. The data denoted with abs represent an absolute value. All methods were performed in accordance with the relevant guidelines and regulations.

### MIDAS

The MIDAS is a self‐reporting instrument that was administered to the patients to measure MIDAS, headache pain intensity, and headache attack frequency. According to the total score (0–21 and more), the severity of the migraine is classified into grades I–IV (I = little or no disability, II = mild disability, III = moderate disability and IV = severe disability)^17^. In the study, the MIDAS score was analysed as a dichotomous variable: low to moderate levels of disability (MIDAS scores = 0–20) and severe levels of disability (MIDAS score ≥ 21).

### HIT-6

The Headache Impact Test-6 (HIT-6) was developed to measure a wide spectrum of factors contributing to the burden of a headache, and it has demonstrated utility for generating quantitative and pertinent information on the impact of a headache. The disability was classified using the following two impact grades based on the HIT-6 score: little to substantial impact (HIT-6 score: 36–59) and severe impact (HIT-6 score: > 60)^18,19^.

### Statistical analysis

A total of 49 EM patients and 50 healthy controls (HCs) were studied. First, we used t-tests (for normally distributed parameters) or a non-parametric Mann–Whitney U test with non-pooled SDs (for non-normally distributed parameters) to compare differences in the mean or median values of 52 lymphocyte characteristics (parameters) between the groups. Lilliefors normality test was used to assess whether the parameter was normally or non-normally distributed. However, for the categorical sex parameter, a chi-square test of independence was used. The Benjamini–Hochberg (B–H) procedure was used to minimise the false discovery rate. The effect size for all numerical parameters (except for sex) was assessed by Cohen's d, which is the standardised difference between group means. Cramér's V is reported for sex.

The relationships between each of the 3 EM parameters (MIDAS score, MIDAS severity and HIT-6) and each of the 52 lymphocyte characteristics within the patient group were examined first by Spearman’s correlation coefficient (rho). Next, we examined the relationships between EM parameters and lymphocyte characteristics using regression analysis. We categorised the numerical HIT-6 outcome as a binary variable with a threshold value of 60. For binary HIT-6 and MIDAS severity outcomes, we ran a series of univariate logistic regression models (with lymphocyte parameters as explanatory variables). The logarithmised MIDAS score outcome was fitted to an OLS regression model. Thereafter, the statistical significance of parameters was validated using an adjusted (multivariate) logistic (OLS) regression model with added covariates (sex and age) to reduce the (latent) possible effect of these covariates on the EM parameters. The goodness of fit of each univariate and multivariate logistic regression model (strength of association) was evaluated by the index of determination (R2) for OLS models and Nagelkerke pseudo-R2 for logistic regression models. Both correlation and regression analyses were performed on a subgroup of 44 EM patients. This group contained 44 patients who all had lymphocyte parameters measured (no missing values for any parameters) and who did not have extreme values. An extreme value was a value that is five times the interquartile range away from the median. All analyses were performed using the statistical software R (www.r-project.org/); the reported p-values are two-tailed, and a 5% significance level was chosen.

## Results

### Characteristics of EM patients and HC

There were 38 (77.6%) females among the EM patients and 37 (74.0%) females among the HCs; therefore, the number of females in each group was nearly identical. The EM patients were 4 years younger on average than the HC patients (EM: mean = 41. 0 (interquartile range = 18); HC: 47.5 (17)). The demographic characteristics are summarised Table 1.

**Table 1:** Characteristics of EM patients and HC.

Characteristic	Patients (49)	Controls (50)	Group comparison	
			P value	Effect Size
Gender—No. Females (%)	38 (77.6%)	37 (74.0%)	0.859	0.024
Age	41 (18)	47.5 (17)	0.186	0.35
Comorbidity—No. (%)	0 (0.0%)	0 (0.0%)	1.000	0.00
Migraine days				
Frequency per month	4.97			
Duration of migraine (years)	16.9			

Age is reported as median (interquartile range). Gender and comorbidity are summarised by frequency and proportion. Effect size is assessed by Cohen's d. All P values (two-sided alternative hypothesis) are reported after a Benjamini–Hochberg correction.

### Comparison of the parameters of the immune system between EM patients and the control group

EM patients differed from healthy controls in several parameters. Table 2 shows the description and comparison of lymphocyte parameters with main changes between EM patients and healthy controls. Supplementary Table 2 shows all the parameters.

**Table 2:** Lymphocyte parameters' description and comparison for EM patients and healthy controls.

Characteristic	Patients (49)	Controls (50)	Groups' comparison	
			P value	Effect Size
CD3 abs^†^	1.99 (1.59)	1.46 (0.74)	0.068	0.716
CD3 LEU (% from leukocytes)	28.88 (18.93)	22.14 (10.63)	0.062	0.687
CD4 + T_EMRA_ (% from CD4 +)^†^	3.45 (5.25)	1.12 (2.76)	0.062	0.588
CD4 + T_EMRA_ abs^†^	42.03 (83.59)	8.65 (29.17)	0.062	0.7
CD8 abs^†^	0.66 (0.49)	0.52 (0.34)	0.062	0.593
CD8 LEU (% from leukocytes)^†^	9.65 (5.51)	7.49 (4.59)	0.062	0.575
CD8 naive abs^†^	265.92 (223.44)	196.42 (175.85)	0.071	0.573
CD19 switched memory abs^†^	48.57 (41.66)	35.88 (22.45)	0.071	0.467
Lymphocytes (% from leukocytes)	39.53 (20.61)	31.56 (12.62)	0.062	0.652

All characteristics are reported median (interquartile range).

^†^Mann–Whitney U test used for non-normally distributed characteristics. Effect size is assessed by Cohen's d. All P values (two-sided alternative hypothesis) are reported after a Benjamini–Hochberg correction.

The groups differed moderately (effect size > 0.6) and with a p-value < 0.10 in 4 parameters: relative count of lymphocytes, relative and absolute number of CD3 T cells, and absolute number of CD4 + T_EMRA_, and the values in the EM patients were clearly higher than those in the healthy controls. In addition, other five parameters, relative number of CD4 + T_EMRA_ and both relative and absolute numbers of CD8 T cells, absolute number of naïve CD8 T cells and the absolute number of CD19 switched memory B cells, have shown changes between EM patients and healthy subjects (Table 2).

### Correlation between immune system parameters and migraine severity

Subsequently, a correlation analysis (Spearman's rho) was performed between each pair of lymphocyte parameters (52 parameters) with paraclinical tests (MIDAS, MIDAS degree and HIT-6). In the MIDAS degree, patients were divided into four groups according to the values of the MIDAS test.

Correlations were evaluated for a subgroup of 44 (out of 49) patients for whom all values were measured and whose values were not extreme (5 × the IQR from the median). Table 3 summarises the statistically significant correlations. Supplementary Table 3 reports all the correlation coefficients.

**Table 3:** All Spearman's correlation coefficients between lymphocyte parameters and migraine outcomes for patients.

Characteristic	MIDAS	Degree of MIDAS	HIT-6
Treg CD45RA + (% of Treg)	0.23 (0.269)	0.25 (0.213)	0.33 (0.087)
CD4 + T_EM_ (% of CD4 +)	− 0.24 (0.228)	− 0.22 (0.281)	− 0.4 (0.027)
CD8 + T_EMRA_ (% of CD8 +)	− 0.09 (0.667)	− 0.08 (0.728)	− 0.41 (0.022)
CD3 + CD57 + (% of CD3 +)	− 0.17 (0.431)	− 0.17 (0.431)	− 0.34 (0.073)

Values in the table are reported Spearman's correlation coefficients and their P values in brackets.

Among the lymphocyte parameters, CD4 + T_EM_ (% of CD4 + ; rho equal to − 0.40) and CD8 + T_EMRA_ (% of CD8 + ; rho equal to − 0.41) were negatively correlated with the HIT-6 at a 5% significance level. A negative correlation indicated that lower values of the HIT-6 occurred with higher values of these parameters. A weak correlation with a p-value < 0.10 was observed between the HIT-6 and 2 parameters, CD3 + CD57 + (% of CD3 +) (− 0.34) and Treg CD45RA + (% of Treg) (0.33).

### Regression modelling of migraine severity

#### MIDAS degree modelling using logistic regression

For MIDAS degree regression modelling, patients were divided into two groups according to the MIDAS test values. The first subgroup included patients with MIDAS values of 1–3 (MIDAS scores = 0–20), and the second subgroup included patients with MIDAS 4 values (MIDAS score ≥ 21). The modelling was performed using logistic regression. Table 4 shows the regression analysis results (95% confidence interval, odds ratio estimates and the pseudo-R2) for the lymphocyte parameters with main changes (p value < 0.1). Supplementary Table 4 reports all the regression results.

**Table 4:** Statistically significant univariate regression models for the strength of migraine assessments and multivariate models with adjustment by covariates: age and gender.

Variable	Univariate regression models					Multivariate regression models				
	BETA	95% LCI BETA	95% UCI BETA	p	R2	BETA	95% LCI BETA	95% UCI BETA	p	R2
**Logistic regression models for MIDAS-degree**										
CD3 + CD57 + (% of CD3 +)	0.92	0.84	1.00	0.058	0.13	0.94	0.85	1.02	0.144	0.17
CD19 non-switched memory abs	1.02	1.00	1.04	0.095	0.09	1.01	0.99	1.04	0.184	0.16
**OLS regression models for MIDAS (values from 0 to 100)**										
CD4 + T_EM_ (% of CD4 +)	0.97	0.93	1.00	0.068	0.08	0.98	0.94	1.02	0.243	0.19
**Logistic regression models for HIT-6**										
CD4 + T_EM_ (% of CD4 +)	0.82	0.69	0.93	0.01	0.33	0.84	0.70	0.95	0.02	0.38
CD3 + CD57 + (% of CD3 +)	0.92	0.85	1	0.062	0.13	0.9	0.8	1	0.058	0.28
Treg (CD4) (% of CD4 +)	0.76	0.55	1	0.065	0.13	0.74	0.5	1.01	0.086	0.26
CD4 naïve (% of CD4 +)	1.07	0.99	1.16	0.098	0.11	1.04	0.96	1.14	0.398	0.18

BETA is the estimated regression coefficient. For logistic regression models, the odds ratio is reported (exponentiated regression coefficient). 95% LCI/UCI BETA is 95% lower and upper confident interval estimates. R2 is the coefficient of determination for OLS model and pseudo coefficient of determination for logistic models.

In the group of patients with a lower MIDAS degree (1, 2 or 3), there were 26 patients (18 (69%) females) with a mean age of 42.9 years, and in the MIDAS degree 4 group, there were 18 patients (16 (88.9%) females) with a mean age of 38.5 years. None of the lymphocyte parameters explained (variability of the values) the MIDAS degree and were statistically significant (Table 4). However, 2 lymphocyte parameters, CD3 + CD57 + (p = 0.058; R2 = 0.13) and CD19 non-switched memory abs (0.095; 0.09), displayed p-values < 0.10. These parameters were not significant at a 10% significance level after adjusting for age and gender. These results (after changing the MIDAS categorisations from 4 to 2 groups) differed from the correlation results. For the 4-degree MIDAS, there were no significant parameters (Table 4).

Neither age nor gender explain the MIDAS degree. As expected, the MIDAS degree was best explained by another parameter of migraine, the HIT-6 (0.027; 0.18).

#### MIDAS 0–100 value modelling by a regression model (OLS)

A MIDAS value in the range of 0–100 explained (“predicted”) with p value < 0.10 1 lymphocyte parameter, CD4 + T_EM_ (% of CD4 +) (0.068; 0.08). Age significantly explained the MIDAS numerical value (0.017; 0.13). The combination of age and NK CD69 + explained 17% of the variability.

#### HIT-6 modelling (categorised as < 60 and >  = 60) using logistic regression

Correlation of four parameters with HIT-6 (as described in correlation analysis results) has p value < 0.10: Treg CD45RA + (% of Treg), CD4 + T_EM_ (% of CD4 +), CD8 + T_EMRA_ (% of CD8 +), and CD3 + CD57 + (% of CD3 +). When the outcome of the HIT-6 was categorised, the study was limited to a low number of patients with HIT-6 values up to 60. (Only 9 patients had HIT-6 values of less than 60, and 35 patients had values of 60 and more, specifically 60–74).

The absolute number of CD45RA + Treg cells for patients with HIT < 60 (25.6 (13.9)) is lower compared to patients with HIT-6 > 60 (35.2 (16.1)) (Supplementary Fig. 4A). On the contrary, CD4 + T_EM_ are statistically significantly (P value = 0.023) augmented in patients with a lower disease severity (HIT-6 < 60; 24.6 (11.4)) vs. severe patients (HIT-6 > 60; 16.4 (9.4)) (Supplementary Fig. 4B). The same (but not statistically significant) happened for CD8 + T_EMRA_ (HIT-6 < 60; 25.1 (18.1) vs. HIT-6 > 60; 21.4 (16.8)), and CD3 + CD57 + (HIT-6 < 60; 24.8 (20.9) vs. HIT-6 > 60; 11.8 (9.0)) (Supplementary Fig. 4C and D).

**Figure 1:**
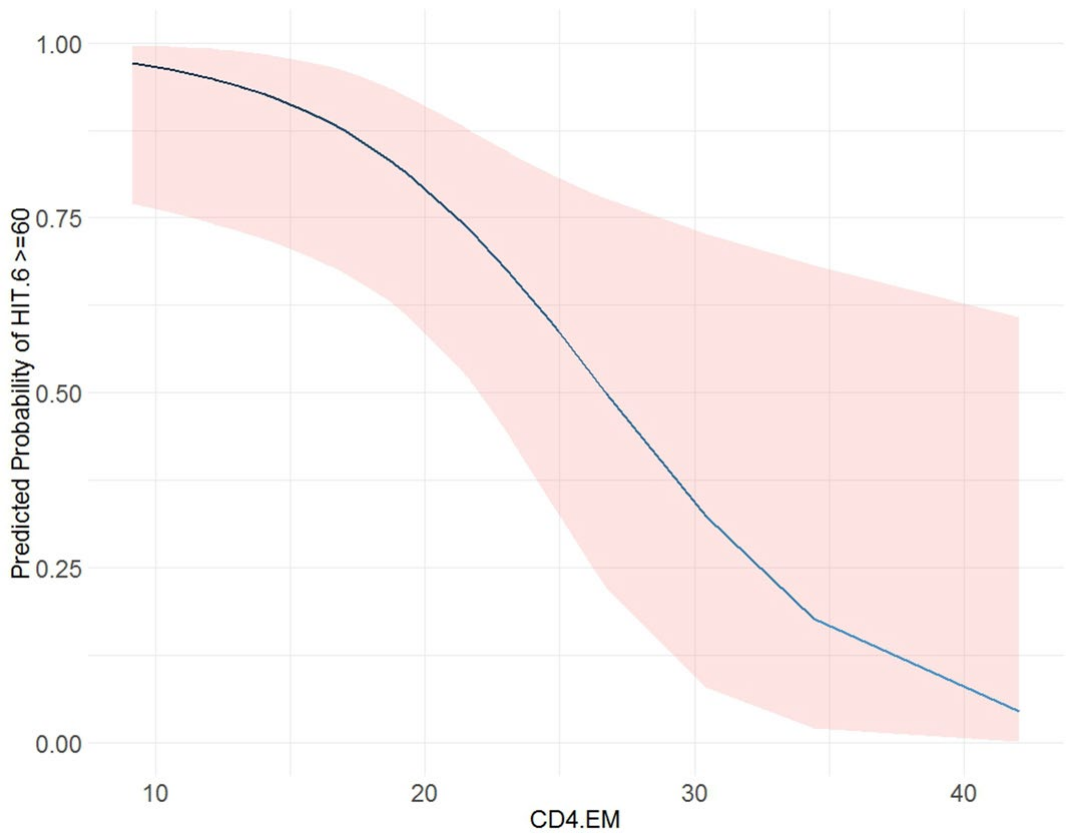
Predicted probability of a HIT-6 value of greater than or equal to 60 based on the CD4 EM values. The blue curve shows the predicted probability (point estimator) of a HIT-6 value of greater than or equal to 60 for the range of observed CD4 EM values. The shaded (light red) area depicts the 95% confidence interval estimate of the probability.

Conclusions on non-identification should be interpreted with caution because of the small number of patients CD4 + T_EM_ values up to 20 predicted a high probability that the HIT-6 > 60 (Fig. 1).

## Discussion

The objective of this study was to evaluate the differences in acquired immunity parameters in patients with EM and in healthy controls. In our study, EM patients showed higher values than healthy controls for nine parameters: the relative count of lymphocytes, relative and absolute counts of CD3 T cells, relative and absolute counts of CD8 suppressor cytotoxic T cells, relative and absolute counts of CD4 + T_EMRA_, absolute count of CD8 naïve T cells, and absolute count of CD19 switched memory B cells respectively.

CD4 + T_EMRA_ are terminally differentiated effector memory lymphocytes. Terminally differentiated helper T lymphocytes have been implicated in protective immunity against pathogens^20^. Significantly higher values of absolute counts of terminally differentiated helper T lymphocytes compared to the healthy controls were found in our work.

CD3 abs represent the absolute value of total T cells. Their increased value is observed in both protective inflammatory response fighting infectious or cancerous diseases and harmful inflammation indicating immunopathological autoimmune diseases. Grazzi et al.^14^ demonstrated that CD3, CD4 and CD19 (expressed as absolute numbers) were significantly higher in patients with chronic migraine those with episodic migraine. In this work, the significant increase in absolute numbers of both CD3 T cells and CD19 B cells was observed in migraine patients compared to results for healthy controls.

CD8 abs represents the absolute value of cytotoxic/suppressor T cells. Resting naïve CD8 T cells have an astounding capacity to react to intracellular pathogens such as viruses via massive expansion and terminal differentiation into cytotoxic effector cells^21^. CD8 T cells are involved in the immunopathogenesis of multiple sclerosis^22^. Cseh et al.^13^ examined lymphocyte levels in paediatric migraine patients and found that the CD8 prevalence was lower in patients than in controls^13^. Covelli et al.^23^ did not observe a change in this peripheral blood parameter in migraine patients^23^. The present study reported an elevation in CD8 abs in migraine patients.

The mechanisms underlying migraine onset are largely unknown. The role of the immune system in migraine onset is a matter of debate. A series of clinical investigators have reported alterations in immune function in migraine patients. However, the results of this work are inconsistent with the findings of other investigators and showed that there were alterations in the immune system function in patients who were treated for EM. Therefore, the next step of this work was to determine whether the changes found in the peripheral blood parameters were related to migraine severity according to the standardised MIDAS and HIT-6 tests in EM patients.

Among the lymphocyte parameters, CD4 + T_EM_ and CD8 + T_EMRA_ were statistically significantly associated with HIT-6. Effector T cells play an important role in immunity against pathogenic agents^24^. Previous studies have noted that subjects with type 2 diabetes mellitus had elevated percentages of effector memory T cells^25^. CD8 + T_EMRA_ exhibit potent effector functions, including the ability to secrete proinflammatory cytokines and cytotoxic molecules^26^.

CD4 + T_EM_ seems to be a significant parameter. The key study by Sallusto and Lanzavecchia^27^ revealed that CD4 + T_EM_ generated more IFNγ^27^. In addition, IFNγ-stimulated dendritic cell-derived exosomes decrease oxidative stress and enhance recovery from a demyelinating damage in slice cultures^28^. Research also indicates that there is an association between myelin integrity and susceptibility to expanding depression^29^. Cortical expanding depression might start intracranial neurogenic inflammation, consequently resulting in migraine headaches via subsequent activation of trigeminal afferents^30–32^. Pusic et al.^33^ demonstrated that interferon gamma-stimulated dendritic cell exosomes (IFNγ-DC-Exos) might decrease susceptibility to expanding depression in vivo and in vitro, which means that IFNγ-DC-Exos might be a possible therapeutic for migraine^33^.

In this study, the most interesting parameter appeared to be already aforementioned CD4 + T_EM_. The HIT-6 migraine outcome was (much) better explained by CD4 + T_EM_ (33%) than by age (15%). On the other hand, age was better described (13% variability explained) by the outcome MIDAS 0–100 than by the best lymphocyte parameter, CD4 + T_EM_ (8%). The combination of CD4 + T_EM_ and age explained 17% of the variability for MIDAS and 37% of the variability for the HIT-6. The results of this work showed that lower CD4 + T_EM_ values mean that there is an increased probability of a MIDAS score ≥ 21. The interval estimate of probability was inaccurate due to the small number of patients in the groups. For this reason, this estimate should be verified in a larger, independent sample.

In relation to the HIT-6, CD4 + T_EM_ appear to be an important parameter. Patients with an CD4 + T_EM_ value below 15 had a high probability (90%) of having a HIT-6 value above 60. This is an estimate of the probability. The interval estimate for this probability was 75–95%. In other words, this probability predicts that 9 out of 10 patients with CD4 + T_EM_ < 15 will have a HIT-6 higher than 60. Results of this study show that CD4 + T_EM_ could be a biomarker that determines the severity of migraine.

Biomarkers can be characterized as physical signs or laboratory measurements connected with a biological process possessing presumed indicative and predicative utility^34^. One can expect that biomarkers associated with the putative pathophysiology of migraine might possess a corresponding value as diagnostic or therapeutic indicators by detecting patients at an increased risk for migraine expansion or by predicting the efficacy of migraine interventions. At present, biomarkers connected with the elevated risk for migraine and biomarkers connected with clinical reactions to treatment are known^35^. Although several circulating biomarkers have been proposed as diagnostic or therapeutic tools in migraine, their identification is still a challenge for the scientific community, reflecting, at least in part, disease complexity and clinical diagnostic limitations.

The biomarkers associated with clinical responses to treatment are interesting for clinical practice. At present, CGRP represents the most promising candidate as a diagnostic and/or therapeutic biomarker as its plasma levels are elevated during a migraine attack and decrease during successful treatment^36^. Cernuda-Morollón et al. have shown that onabotulinumtoxinA significantly decreases CGRP levels in peripheral blood samples between attacks and measured 1 month after injection, thus confirming interictal, peripheral levels of CGRP as a potential chronic migraine biomarker^37^. Other treatment response biomarkers may be neurokinin A and serotonin transporter. Neurokinin A levels measured before drug administration were significantly higher in responders vs nonresponders to rizatriptan^38^. STin 2.12/12 genotype was identified as a significant factor increasing the odds for an inconsistent response to triptans^39^. CD4 + T_EM_ may be a new biomarker, one that assesses the severity of migraine in patients without prophylactic treatment. As new prophylactic anti-migraine treatments are now available (anti-CGRP monoclonal antibodies), it is important to identify a patient early with a more severe course of migraine so that therapy is rationally selected for each patient in an effort to increase their quality of life as much as possible.

Peripheral blood immune parameters were evaluated in this study. We are aware that absolute lymphocyte counts are highly variable and may considerably change even during a day. For this reason, samples were taken approximately at the same time. We are also aware that migraineurs often suffer from comorbid atopic disorders and are exposed to chronic stress due to frequent headaches, and this stress is the cause of prolonged release of endogenous corticosteroids to cope with the potentially harmful consequences of this stress. However, the price of this regulatory effort is dampening protective capacities of the immune system. To eliminate the factors that could affect the immune system, only patients without known comorbidities and for whom migraine was their only disease were selected for this analysis.

In spite of that we have chosen 5% statistical significance level we interpret the non-significant results with p value < 0.10 because we studied many lymphocyte parameters in a relatively small sample of EM patients. The use of a high number of variables implies that by chance some of the measurements will display statistical significance. This is the reason for using a statistical test with correction for multiple comparisons. The statistical power of these tests is lower because they are much more restrictive, and this is the reason why we did not get statistical significance (p < 0.05) in the variables measured. We focused on describing our data set in order to identify a few potential biomarkers from the set of many parameters because there are no studies investigating these relationships. Our findings should be validated by another study.

## Conclusions

The results of this study show that EM patients show changes in immune system parameters as determined by peripheral blood collection. CD4 + T_EM_ appears to be an important parameter. Lower CD4 + T_EM_ values mean that there is an increased probability of a severe disability. The CD4 + T_EM_ may represent a potential biomarker that determines the severity of migraine. However, these results should be validated in a larger cohort of EM patients.

## Supplementary information

Supplementary file1 (DOCX 818 kb)

## Abbreviations

EM: Episodic migraine
IFNγ: Interferon gamma
CGRP: Calcitonin gene-related peptide
CD4 + T_EM_: Effector memory helper T lymphocytes
CD8 + T_EMRA_: Terminally differentiated cytotoxic T lymphocytes
CD4 + T_EMRA_: Terminally differentiated helper T lymphocytes
