# Supplementary material for: The role of the immune system and the biomarker CD3 + CD4 + CD45RA−CD62L− in the pathophysiology of migraine
Source: Sci Rep. 2020 Jul 23;10:12277. doi: 10.1038/s41598-020-69285-4 (PMC7378179; doi:10.1038/s41598-020-69285-4)
Supplement: Supplementary file 1 — Supplementary file1 (DOCX 818 kb) [file 41598_2020_69285_MOESM1_ESM.docx]

**The role of the immune system and the biomarker CD3+CD4+CD45RA-CD62L- in the pathophysiology of migraine**

Zbyšek Pavelek, MD, PhD^1*^, Ondřej Souček^2^, Jan Krejsek, MD, PhD^2^, Lukáš Sobíšek, PhD^1^, Blanka Klímová, PhD^1^, Jiří Masopust, MD, PhD^1^, Kamil Kuča, PhD^3^, Martin Vališ, MD, PhD^1^

^1^ Department of Neurology, Faculty of Medicine and University Hospital Hradec Králové, Charles University in Prague, Hradec Králové, Czech Republic

^2^ Department of Clinical Immunology and Allergology, University Hospital Hradec Králové, Hradec Králové, Czech Republic

^3^Biomedical Research Center, University Hospital Hradec Kralové, Hradec Kralové, Czech Republic

***Corresponding author**

Zbyšek Pavelek

Department of Neurology, Faculty of Medicine and University Hospital Hradec Králové, Charles University in Prague, Hradec Králové, Sokolská 581, 500 05, Czech Republic

Tel. +420495835251

Fax +420495835216

Email: [zbysekpavelek@email.cz](mailto:zbysekpavelek@email.cz)

**Suppl. Table 1.** – Combination of fluorochrome-conjugated antibodies used for the immunophenotypic analysis of lymphocytes from blood samples by flow cytometry

Tube no. 1 – T cells markers

| **Fluorochrome conjugated to antibody reagent** | **FITC** | **PE** | **PC5.5** | **PC7** | **APC** | **APC-a750** | **PB** | **KrO** |
| --- | --- | --- | --- | --- | --- | --- | --- | --- |
| **Antibody marker** | CD45-RA | CD62L | CD4 | CD127 | CD8 | CD3 | CD25 | CD45 |
| **Antibody clone** | ALB11 | DREG56 | 13B8.2 | R34.34 | B9.11 | UCHT1 | B1.49.9 | J33 |
| **Antibody manufacturer** | Beckman Coulter | Beckman Coulter | Beckman Coulter | Beckman Coulter | Beckman Coulter | Beckman Coulter | Beckman Coulter | Beckman Coulter |

Tube no. 2 – NK cells markers and markers of activation and exhaustion

| **Fluorochrome conjugated to antibody reagent** | **FITC** | **PE** | **PC7** | **APC** | **APC-a750** | **PB** | **KrO** |
| --- | --- | --- | --- | --- | --- | --- | --- |
| **Antibody marker** | CD16 | CD69 | CD19 | CD56 | CD3 | CD57 | CD45 |
| **Antibody clone** | 3G8 | TP1.55.3 | J3-119 | N901 (NKH-1) | UCHT1 | NC1 | J33 |
| **Antibody manufacturer** | Beckman Coulter | Beckman Coulter | Beckman Coulter | Beckman  Coulter | Beckman  Coulter | Beckman Coulter | Beckman Coulter |

Tube no. 3 – B cells markers

| **Fluorochrome conjugated to antibody reagent** | **FITC** | **PE** | **PC7** | **KrO** |
| --- | --- | --- | --- | --- |
| **Antibody marker** | CD27 | IgD | CD19 | CD45 |
| **Antibody clone** | M-T271 | polyclonal rabbit F(ab´)2 | J3-119 | J33 |
| **Antibody manufacturer** | Dako | Dako | Beckman Coulter | Beckman Coulter |

Legend: APC, Allophycocyanin; *APC-a750*, Alexa Fluor 750; FITC, fluorescein isothiocyanate; *KrO*, Krome Orange PE, phycoerythrin; *PC5*, phycoerythrin-cyanin 5.5; *PC7*, phycoerythrin-cyanin 7; *PB*, Pacific Blue

**Suppl. Table 2.** Description and comparison of all of the characteristics of EM patients and healthy controls

| **Characteristic** | **Patients (49)** | **Controls (50)** | **Groups' comparison** | |
| --- | --- | --- | --- | --- |
|  |  |  | **P value** | **Effect Size** |
| Gender - No. Females (%) | 38 (77.6%) | 37 (74.0%) | 0.859 | 0.024 |
| Age | 41 (18) | 47.5 (17) | 0.186 | 0.35 |
| CD3 abs | 1.99 (1.59) | 1.46 (0.74) | 0.068 | 0.716 |
| CD3 LEU (% from leukocytes) | 28.88 (18.93) | 22.14 (10.63) | 0.062 | 0.687 |
| CD3 LYMFO (% from lymphocytes) | 72.81 (12.28) | 72.77 (12.04) | 0.34 | 0.266 |
| CD3+CD57+ (% from CD3+) | 11.8 (10.5) | 11.25 (12.95) | 0.992 | 0.003 |
| CD3+CD57+ (abs) | 0.24 (0.25) | 0.19 (0.21) | 0.497 | 0.276 |
| CD3+CD69+ (% from CD3+) | 6.99 (3.08) | 5.74 (3.07) | 0.159 | 0.289 |
| CD4 abs | 1.04 (0.9) | 0.9 (0.45) | 0.458 | 0.483 |
| CD4+ T_CM_ (% from CD4+) | 36.21 (12.08) | 37.56 (11.33) | 0.589 | 0.204 |
| CD4+ T_CM_ abs | 339.19 (252.16) | 322.37 (120.38) | 0.693 | 0.376 |
| CD4+ T_EM_ (% from CD4+) | 18.66 (9.94) | 16.31 (8.12) | 0.333 | 0.276 |
| CD4+ T_EM_ abs | 205.5 (195.26) | 134.65 (111.72) | 0.159 | 0.507 |
| CD4 LEU (% from leukocytes) | 14.32 (11.34) | 12.9 (7.14) | 0.491 | 0.417 |
| CD4 LYMFO (% from lymphocytes) | 45.26 (11.45) | 46 (12.59) | 0.82 | 0.115 |
| CD4 naive (% from CD4+) | 38.74 (8.31) | 43.13 (18.83) | 0.375 | 0.24 |
| CD4 naive abs | 390.11 (400.78) | 372.26 (271.28) | 0.618 | 0.337 |
| CD4+ T_EMRA_ (% from CD4+) | 3.45 (5.25) | 1.12 (2.76) | 0.062 | 0.588 |
| CD4+ T_EMRA_ abs | 42.03 (83.59) | 8.65 (29.17) | 0.062 | 0.7 |
| CD8 abs | 0.66 (0.49) | 0.52 (0.34) | 0.062 | 0.593 |
| CD8+ T_CM_ (% from CD8+) | 12.59 (7.44) | 14.13 (10.66) | 0.693 | 0.092 |
| CD8+ T_CM_ abs | 88.98 (78.06) | 69.05 (45.6) | 0.159 | 0.444 |
| CD8+ T_EM_ (% from CD8+) | 18.75 (10.91) | 20.58 (11.77) | 0.931 | 0.027 |
| CD8+ T_EM_ abs | 99.56 (65.38) | 83.1 (92.37) | 0.178 | 0.396 |
| CD8 LEU (% from leukocytes) | 9.65 (5.51) | 7.49 (4.59) | 0.062 | 0.575 |
| CD8 LYMFO (% from lymphocytes) | 27.46 (8.55) | 24.39 (8.33) | 0.166 | 0.386 |
| CD8 naive (% from CD8+) | 38.71 (18.33) | 40.52 (23.54) | 0.961 | 0.03 |
| CD8 naive abs | 265.92 (223.44) | 196.42 (175.85) | 0.071 | 0.573 |
| CD8+ T_EMRA_ (% from CD8+) | 24.1 (20.04) | 22.49 (14.96) | 0.916 | 0.076 |
| CD8+ T_EMRA_ abs | 160.38 (206.32) | 116.35 (122.42) | 0.166 | 0.41 |
| CD19 abs | 0.28 (0.19) | 0.23 (0.12) | 0.215 | 0.421 |
| CD19 LEU (% from leukocytes) | 4.28 (2.61) | 3.84 (2.11) | 0.262 | 0.458 |
| CD19 LYMFO (% from lymphocytes) | 10.91 (4.45) | 11.3 (5.23) | 0.89 | 0.126 |
| CD19 memory CD27- (% from B lymphocytes) | 3.4 (3.05) | 3.16 (2.96) | 0.943 | 0.017 |
| CD19 memory CD27- abs | 8.73 (8.82) | 6.37 (7.57) | 0.459 | 0.273 |
| CD19 naive (% from B lymphocytes) | 63.64 (12.9) | 64.81 (18.64) | 0.916 | 0.031 |
| CD19 naive abs | 175.21 (127.48) | 150.79 (84.06) | 0.316 | 0.37 |
| CD19 nonswitched memory (% from B lymphocytes) | 16.11 (8.72) | 14.38 (8.93) | 0.915 | 0.168 |
| CD19 nonswitched memory abs | 44.33 (38.03) | 35.18 (27.14) | 0.352 | 0.081 |
| CD19 switched memory (% from B lymphocytes) | 16.75 (8.09) | 15.16 (9) | 0.497 | 0.166 |
| CD19 switched memory abs | 48.57 (41.66) | 35.88 (22.45) | 0.071 | 0.467 |
| CD19+CD69+ (% from CD19+) | 2.66 (1.4) | 2.62 (2.32) | 0.916 | 0.294 |
| LEUKO | 6.63 (2.1) | 6.52 (2.59) | 0.82 | 0.047 |
| LYMPHO | 2.05 (0.74) | 2.02 (0.83) | 0.992 | 0.064 |
| Lymphocytes (% from leukocytes) | 39.53 (20.61) | 31.56 (12.62) | 0.062 | 0.652 |
| NK abs | 0.37 (0.22) | 0.31 (0.2) | 0.34 | 0.121 |
| NK CD57+ (% from NK) | 30.89 (16.96) | 41.27 (18.15) | 0.172 | 0.337 |
| NK CD57+ abs | 0.13 (0.13) | 0.12 (0.13) | 0.943 | 0.106 |
| NK CD69+ (% from NK) | 12.46 (14.7) | 10.58 (7.15) | 0.166 | 0.475 |
| NK LEU (% from leukocytes) | 5.45 (3.6) | 4.74 (2.96) | 0.525 | 0.033 |
| NK LYMFO (% from lymphocytes) | 13.97 (10.23) | 14.55 (10.73) | 0.77 | 0.219 |
| Treg (CD4) (% from CD4+) | 5.85 (4.2) | 7.24 (3.38) | 0.375 | 0.237 |
| Treg abs | 0.06 (0.06) | 0.06 (0.03) | 0.992 | 0.238 |
| Treg CD45RA+ (% from Treg) | 34.81 (16.7) | 35.16 (11.59) | 0.82 | 0.06 |
| MIDAS | 16 (21) | na | na | na |
| degree of MIDAS | 3 (2) | na | na | na |
| HIT-6 | 64 (6) | na | na | na |

Legend: All characteristics are reported median (interquartile range), except Gender. Gender is summarized by frequency and proportion of Female. Effect size is assessed by Cohen's D. All P values (two-sided alternative hypothesis) are reported after Benjamini-Hochberg correction.

**Suppl. Table 3.** All Spearman's correlation coefficients between lymphocyte parameters and migraine outcomes for patients (44).

| **Characteristic** | **MIDAS** | **degree of MIDAS** | **HIT-6** |
| --- | --- | --- | --- |
| Age | -0.32 (0.101) | -0.3 (0.123) | -0.21 (0.312) |
| LEUKO | 0 (0.989) | 0.06 (0.788) | -0.02 (0.914) |
| LYMPHO | 0.05 (0.813) | 0.11 (0.629) | 0.09 (0.663) |
| Lymphocytes (% of leukocytes) | 0.22 (0.295) | 0.28 (0.153) | 0.15 (0.49) |
| CD3 LEU (% of leukocytes) | 0.22 (0.296) | 0.27 (0.182) | 0.16 (0.44) |
| CD3 abs | 0.19 (0.374) | 0.25 (0.223) | 0.14 (0.527) |
| CD19 LEU (% of leukocytes) | 0.18 (0.382) | 0.25 (0.212) | 0.16 (0.466) |
| CD19 abs | 0.13 (0.557) | 0.2 (0.329) | 0.07 (0.761) |
| NK LEU (% of leukocytes) | 0.09 (0.691) | 0.18 (0.408) | -0.02 (0.943) |
| NK abs | 0.1 (0.644) | 0.19 (0.381) | -0.01 (0.949) |
| CD3 LYMFO (% of lymphocytes) | 0.11 (0.61) | 0.07 (0.759) | 0.14 (0.532) |
| CD19 LYMFO (% of lymphocytes) | -0.1 (0.641) | -0.08 (0.723) | -0.1 (0.641) |
| NK LYMFO (% of lymphocytes) | -0.09 (0.689) | -0.03 (0.898) | -0.16 (0.459) |
| CD3+CD57+ (% of CD3+) | -0.17 (0.431) | -0.17 (0.431) | -0.34 (0.073) |
| CD3+CD57+ (abs) | -0.03 (0.913) | 0.01 (0.97) | -0.23 (0.253) |
| NK CD57+ (% of NK) | -0.08 (0.732) | -0.05 (0.832) | -0.18 (0.39) |
| NK CD57+ abs | 0.01 (0.949) | 0.1 (0.658) | -0.07 (0.743) |
| CD3+CD69+ (% of CD3+) | 0.09 (0.676) | 0.09 (0.689) | -0.1 (0.635) |
| CD19+CD69+ (% of CD19+) | 0.05 (0.819) | 0.01 (0.969) | -0.11 (0.601) |
| NK CD69+ (% of NK) | 0 (0.988) | 0.01 (0.97) | 0.08 (0.73) |
| CD4 LEU (% of leukocytes) | 0.18 (0.382) | 0.22 (0.283) | 0.23 (0.252) |
| CD4 abs | 0.15 (0.497) | 0.21 (0.313) | 0.15 (0.478) |
| CD8 LEU (% of leukocytes) | 0.22 (0.287) | 0.28 (0.155) | 0.08 (0.704) |
| CD8 abs | 0.18 (0.387) | 0.28 (0.166) | 0.06 (0.799) |
| CD4 LYMFO (% of lymphocytes) | 0.05 (0.832) | 0.03 (0.898) | 0.2 (0.326) |
| CD8 LYMFO (% of lymphocytes) | 0.1 (0.659) | 0.12 (0.569) | -0.04 (0.855) |
| Treg (CD4) (% of CD4+) | 0.14 (0.504) | 0.21 (0.298) | -0.01 (0.968) |
| Treg abs | 0.15 (0.47) | 0.23 (0.268) | 0.09 (0.662) |
| Treg CD45RA+ (% of Treg) | 0.23 (0.269) | 0.25 (0.213) | 0.33 (0.087) |
| CD4+ T_CM_ (% of CD4+) | -0.11 (0.61) | -0.17 (0.432) | -0.02 (0.919) |
| CD4+ T_CM_ abs | 0.1 (0.654) | 0.12 (0.587) | 0.23 (0.268) |
| CD4 naive (% of CD4+) | 0.04 (0.845) | 0.12 (0.587) | 0.13 (0.555) |
| CD4 naive abs | 0.16 (0.441) | 0.22 (0.287) | 0.2 (0.331) |
| CD4+ T_EMRA_ (% of CD4+) | 0.08 (0.704) | 0.19 (0.353) | -0.06 (0.795) |
| CD4+ T_EMRA_ abs | 0.1 (0.645) | 0.2 (0.34) | -0.01 (0.949) |
| CD4+ T_EM_ (% of CD4+) | -0.24 (0.228) | -0.22 (0.281) | -0.4 (0.027) |
| CD4+ T_EM_ abs | 0.01 (0.964) | 0.06 (0.788) | -0.07 (0.74) |
| CD8+ T_CM_ (% of CD8+) | -0.06 (0.796) | -0.11 (0.616) | 0.16 (0.463) |
| CD8+ T_CM_ abs | 0.18 (0.386) | 0.22 (0.285) | 0.24 (0.231) |
| CD8 naive (% of CD8+) | 0.06 (0.788) | 0.11 (0.612) | 0.29 (0.138) |
| CD8 naive abs | 0.17 (0.431) | 0.26 (0.203) | 0.21 (0.3) |
| CD8+ T_EMRA_ (% of CD8+) | -0.09 (0.667) | -0.08 (0.728) | -0.41 (0.022) |
| CD8+ T_EMRA_ abs | 0.13 (0.564) | 0.19 (0.367) | -0.2 (0.329) |
| CD8+ T_EM_ (% of CD8+) | 0.1 (0.643) | 0.02 (0.916) | 0.1 (0.647) |
| CD8+ T_EM_ abs | 0.26 (0.203) | 0.26 (0.191) | 0.13 (0.546) |
| CD19 naive (% of B lymphocytes) | -0.1 (0.632) | -0.11 (0.607) | -0.2 (0.34) |
| CD19 naive abs | 0.03 (0.891) | 0.12 (0.575) | -0.04 (0.853) |
| CD19 non-switched memory (% of B lymphocytes) | 0.13 (0.537) | 0.12 (0.587) | 0.11 (0.611) |
| CD19 non-switched memory abs | 0.26 (0.208) | 0.28 (0.157) | 0.2 (0.345) |
| CD19 switched memory (% of B lymphocytes) | 0.18 (0.388) | 0.19 (0.381) | 0.21 (0.302) |
| CD19 switched memory abs | 0.24 (0.243) | 0.3 (0.122) | 0.18 (0.393) |
| CD19 memory CD27- (% of B lymphocytes) | -0.12 (0.572) | -0.12 (0.593) | 0.13 (0.555) |
| CD19 memory CD27- abs | -0.07 (0.734) | -0.06 (0.796) | 0.18 (0.399) |

Legend: Spearman's correlation coefficients and their P values are reported in brackets.

**Suppl. Table 3.** All Spearman's correlation coefficients between lymphocyte parameters and migraine outcomes for patients (44).

| **Characteristic** | **MIDAS** | **degree of MIDAS** | **HIT-6** |
| --- | --- | --- | --- |
| Age | -0.32 (0.101) | -0.3 (0.123) | -0.21 (0.312) |
| LEUKO | 0 (0.989) | 0.06 (0.788) | -0.02 (0.914) |
| LYMPHO | 0.05 (0.813) | 0.11 (0.629) | 0.09 (0.663) |
| Lymphocytes (% of leukocytes) | 0.22 (0.295) | 0.28 (0.153) | 0.15 (0.49) |
| CD3 LEU (% of leukocytes) | 0.22 (0.296) | 0.27 (0.182) | 0.16 (0.44) |
| CD3 abs | 0.19 (0.374) | 0.25 (0.223) | 0.14 (0.527) |
| CD19 LEU (% of leukocytes) | 0.18 (0.382) | 0.25 (0.212) | 0.16 (0.466) |
| CD19 abs | 0.13 (0.557) | 0.2 (0.329) | 0.07 (0.761) |
| NK LEU (% of leukocytes) | 0.09 (0.691) | 0.18 (0.408) | -0.02 (0.943) |
| NK abs | 0.1 (0.644) | 0.19 (0.381) | -0.01 (0.949) |
| CD3 LYMFO (% of lymphocytes) | 0.11 (0.61) | 0.07 (0.759) | 0.14 (0.532) |
| CD19 LYMFO (% of lymphocytes) | -0.1 (0.641) | -0.08 (0.723) | -0.1 (0.641) |
| NK LYMFO (% of lymphocytes) | -0.09 (0.689) | -0.03 (0.898) | -0.16 (0.459) |
| CD3+CD57+ (% of CD3+) | -0.17 (0.431) | -0.17 (0.431) | -0.34 (0.073) |
| CD3+CD57+ (abs) | -0.03 (0.913) | 0.01 (0.97) | -0.23 (0.253) |
| NK CD57+ (% of NK) | -0.08 (0.732) | -0.05 (0.832) | -0.18 (0.39) |
| NK CD57+ abs | 0.01 (0.949) | 0.1 (0.658) | -0.07 (0.743) |
| CD3+CD69+ (% of CD3+) | 0.09 (0.676) | 0.09 (0.689) | -0.1 (0.635) |
| CD19+CD69+ (% of CD19+) | 0.05 (0.819) | 0.01 (0.969) | -0.11 (0.601) |
| NK CD69+ (% of NK) | 0 (0.988) | 0.01 (0.97) | 0.08 (0.73) |
| CD4 LEU (% of leukocytes) | 0.18 (0.382) | 0.22 (0.283) | 0.23 (0.252) |
| CD4 abs | 0.15 (0.497) | 0.21 (0.313) | 0.15 (0.478) |
| CD8 LEU (% of leukocytes) | 0.22 (0.287) | 0.28 (0.155) | 0.08 (0.704) |
| CD8 abs | 0.18 (0.387) | 0.28 (0.166) | 0.06 (0.799) |
| CD4 LYMFO (% of lymphocytes) | 0.05 (0.832) | 0.03 (0.898) | 0.2 (0.326) |
| CD8 LYMFO (% of lymphocytes) | 0.1 (0.659) | 0.12 (0.569) | -0.04 (0.855) |
| Treg (CD4) (% of CD4+) | 0.14 (0.504) | 0.21 (0.298) | -0.01 (0.968) |
| Treg abs | 0.15 (0.47) | 0.23 (0.268) | 0.09 (0.662) |
| Treg CD45RA+ (% of Treg) | 0.23 (0.269) | 0.25 (0.213) | 0.33 (0.087) |
| CD4+ T_CM_ (% of CD4+) | -0.11 (0.61) | -0.17 (0.432) | -0.02 (0.919) |
| CD4+ T_CM_ abs | 0.1 (0.654) | 0.12 (0.587) | 0.23 (0.268) |
| CD4 naive (% of CD4+) | 0.04 (0.845) | 0.12 (0.587) | 0.13 (0.555) |
| CD4 naive abs | 0.16 (0.441) | 0.22 (0.287) | 0.2 (0.331) |
| CD4+ T_EMRA_ (% of CD4+) | 0.08 (0.704) | 0.19 (0.353) | -0.06 (0.795) |
| CD4+ T_EMRA_ abs | 0.1 (0.645) | 0.2 (0.34) | -0.01 (0.949) |
| CD4+ T_EM_ (% of CD4+) | -0.24 (0.228) | -0.22 (0.281) | -0.4 (0.027) |
| CD4+ T_EM_ abs | 0.01 (0.964) | 0.06 (0.788) | -0.07 (0.74) |
| CD8+ T_CM_ (% of CD8+) | -0.06 (0.796) | -0.11 (0.616) | 0.16 (0.463) |
| CD8+ T_CM_ abs | 0.18 (0.386) | 0.22 (0.285) | 0.24 (0.231) |
| CD8 naive (% of CD8+) | 0.06 (0.788) | 0.11 (0.612) | 0.29 (0.138) |
| CD8 naive abs | 0.17 (0.431) | 0.26 (0.203) | 0.21 (0.3) |
| CD8+ T_EMRA_ (% of CD8+) | -0.09 (0.667) | -0.08 (0.728) | -0.41 (0.022) |
| CD8+ T_EMRA_ abs | 0.13 (0.564) | 0.19 (0.367) | -0.2 (0.329) |
| CD8+ T_EM_ (% of CD8+) | 0.1 (0.643) | 0.02 (0.916) | 0.1 (0.647) |
| CD8+ T_EM_ abs | 0.26 (0.203) | 0.26 (0.191) | 0.13 (0.546) |
| CD19 naive (% of B lymphocytes) | -0.1 (0.632) | -0.11 (0.607) | -0.2 (0.34) |
| CD19 naive abs | 0.03 (0.891) | 0.12 (0.575) | -0.04 (0.853) |
| CD19 non-switched memory (% of B lymphocytes) | 0.13 (0.537) | 0.12 (0.587) | 0.11 (0.611) |
| CD19 non-switched memory abs | 0.26 (0.208) | 0.28 (0.157) | 0.2 (0.345) |
| CD19 switched memory (% of B lymphocytes) | 0.18 (0.388) | 0.19 (0.381) | 0.21 (0.302) |
| CD19 switched memory abs | 0.24 (0.243) | 0.3 (0.122) | 0.18 (0.393) |
| CD19 memory CD27- (% of B lymphocytes) | -0.12 (0.572) | -0.12 (0.593) | 0.13 (0.555) |
| CD19 memory CD27- abs | -0.07 (0.734) | -0.06 (0.796) | 0.18 (0.399) |

Legend: Spearman's correlation coefficients and their P values are reported in brackets.

**Suppl. Table 4.** All univariate regression models for the strength of migraine assessments and multivariate models with adjustment for the covariates age and gender

| Variable | Univariate regression models | | | | | Multivariate regression models | | | | |
| --- | --- | --- | --- | --- | --- | --- | --- | --- | --- | --- |
|  | BETA | 95% LCI BETA | 95% UCI BETA | p | R2 | BETA | 95% LCI BETA | 95% UCI BETA | p | R2 |
| ***Logistic regression models for the MIDAS-degree*** | | | | | | | | | | |
| LEUKO | 1.10 | 0.76 | 1.60 | 0.617 | 0.01 | 1.08 | 0.73 | 1.61 | 0.704 | 0.11 |
| LYMPHO | 1.07 | 0.40 | 2.85 | 0.887 | 0.00 | 0.99 | 0.33 | 2.83 | 0.989 | 0.11 |
| Lymphocytes (% of leukocytes) | 1.02 | 0.98 | 1.07 | 0.322 | 0.03 | 1.02 | 0.97 | 1.07 | 0.459 | 0.12 |
| CD3 LEU (% of leukocytes) | 1.02 | 0.97 | 1.08 | 0.392 | 0.02 | 1.02 | 0.96 | 1.07 | 0.584 | 0.11 |
| CD3 abs | 1.52 | 0.79 | 3.05 | 0.215 | 0.05 | 1.40 | 0.69 | 2.90 | 0.346 | 0.13 |
| CD19 LEU (% of leukocytes) | 1.22 | 0.90 | 1.71 | 0.217 | 0.05 | 1.18 | 0.85 | 1.65 | 0.314 | 0.13 |
| CD19 abs | 19.18 | 0.33 | 1755.97 | 0.168 | 0.06 | 12.14 | 0.15 | 1080.03 | 0.257 | 0.14 |
| NK LEU (% of leukocytes) | 1.05 | 0.85 | 1.32 | 0.640 | 0.01 | 1.08 | 0.86 | 1.37 | 0.520 | 0.12 |
| NK abs | 2.42 | 0.12 | 51.14 | 0.557 | 0.01 | 2.72 | 0.13 | 65.64 | 0.520 | 0.12 |
| CD3 LYMFO (% of lymphocytes) | 1.01 | 0.93 | 1.10 | 0.881 | 0.00 | 0.98 | 0.89 | 1.08 | 0.691 | 0.11 |
| CD19 LYMFO (% of lymphocytes) | 1.01 | 0.83 | 1.23 | 0.947 | 0.00 | 1.02 | 0.83 | 1.26 | 0.857 | 0.11 |
| NK LYMFO (% of lymphocytes) | 0.99 | 0.91 | 1.08 | 0.851 | 0.00 | 1.02 | 0.92 | 1.13 | 0.738 | 0.11 |
| CD3+CD57+ (% of CD3+) | 0.92 | 0.84 | 1.00 | 0.058 | 0.13 | 0.94 | 0.85 | 1.02 | 0.144 | 0.17 |
| CD3+CD57+ (abs) | 0.21 | 0.01 | 3.25 | 0.313 | 0.04 | 0.37 | 0.01 | 6.76 | 0.530 | 0.12 |
| NK CD57+ (% of NK) | 0.98 | 0.94 | 1.02 | 0.304 | 0.03 | 0.99 | 0.95 | 1.03 | 0.629 | 0.11 |
| NK CD57+ abs | 0.10 | 0.00 | 17.17 | 0.416 | 0.02 | 0.21 | 0.00 | 41.05 | 0.586 | 0.11 |
| CD3+CD69+ (% of CD3+) | 1.06 | 0.83 | 1.35 | 0.662 | 0.01 | 1.06 | 0.82 | 1.37 | 0.636 | 0.11 |
| CD19+CD69+ (% of CD19+) | 0.79 | 0.46 | 1.26 | 0.342 | 0.03 | 0.66 | 0.32 | 1.17 | 0.195 | 0.16 |
| NK CD69+ (% of NK) | 1.00 | 0.93 | 1.07 | 0.978 | 0.00 | 0.99 | 0.92 | 1.06 | 0.763 | 0.11 |
| CD4 LEU (% of leukocytes) | 1.03 | 0.96 | 1.11 | 0.449 | 0.02 | 1.02 | 0.94 | 1.10 | 0.595 | 0.11 |
| CD4 abs | 1.75 | 0.71 | 4.68 | 0.234 | 0.04 | 1.62 | 0.61 | 4.34 | 0.322 | 0.13 |
| CD8 LEU (% of leukocytes) | 1.03 | 0.90 | 1.18 | 0.664 | 0.01 | 1.02 | 0.88 | 1.18 | 0.750 | 0.11 |
| CD8 abs | 2.48 | 0.38 | 18.71 | 0.349 | 0.03 | 2.26 | 0.28 | 18.50 | 0.433 | 0.12 |
| CD4 LYMFO (% of lymphocytes) | 1.00 | 0.93 | 1.08 | 0.998 | 0.00 | 0.98 | 0.90 | 1.06 | 0.676 | 0.11 |
| CD8 LYMFO (% of lymphocytes) | 1.03 | 0.95 | 1.12 | 0.547 | 0.01 | 1.03 | 0.94 | 1.13 | 0.508 | 0.12 |
| Treg (CD4) (% of CD4+) | 1.10 | 0.88 | 1.38 | 0.412 | 0.02 | 1.21 | 0.94 | 1.59 | 0.148 | 0.17 |
| Treg abs | 7549.30 | 0.20 | 5440178421.24 | 0.14 | 0.08 | 16619.63 | 0.50 | 3282062647.11 | 0.074 | 0.20 |
| Treg CD45RA+ (% of Treg) | 1.03 | 0.99 | 1.09 | 0.141 | 0.07 | 1.02 | 0.98 | 1.08 | 0.353 | 0.13 |
| CD4+ T_CM_ (% of CD4+) | 1.01 | 0.94 | 1.07 | 0.841 | 0.00 | 1.04 | 0.96 | 1.12 | 0.345 | 0.13 |
| CD4+ T_CM_ abs | 1.00 | 1.00 | 1.00 | 0.284 | 0.04 | 1.00 | 1.00 | 1.00 | 0.208 | 0.15 |
| CD4 naive (% of CD4+) | 1.01 | 0.95 | 1.06 | 0.808 | 0.00 | 0.98 | 0.92 | 1.04 | 0.567 | 0.12 |
| CD4 naive abs | 1.00 | 1.00 | 1.00 | 0.334 | 0.03 | 1.00 | 1.00 | 1.00 | 0.714 | 0.11 |
| CD4+ T_EMRA_ (% of CD4+) | 1.03 | 0.91 | 1.18 | 0.623 | 0.01 | 1.03 | 0.90 | 1.18 | 0.690 | 0.11 |
| CD4+ T_EMRA_ abs | 1.00 | 1.00 | 1.01 | 0.386 | 0.02 | 1.00 | 1.00 | 1.01 | 0.392 | 0.13 |
| CD4+ T_EM_ (% of CD4+) | 0.92 | 0.82 | 1.01 | 0.110 | 0.09 | 0.93 | 0.82 | 1.03 | 0.212 | 0.15 |
| CD4+ T_EM_ abs | 1.00 | 1.00 | 1.00 | 0.988 | 0.00 | 1.00 | 0.99 | 1.01 | 0.939 | 0.11 |
| CD8+ T_CM_ (% of CD8+) | 1.04 | 0.95 | 1.14 | 0.374 | 0.02 | 1.08 | 0.97 | 1.21 | 0.168 | 0.16 |
| CD8+ T_CM_ abs | 1.01 | 1.00 | 1.03 | 0.119 | 0.08 | 1.01 | 1.00 | 1.03 | 0.069 | 0.20 |
| CD8 naive (% of CD8+) | 1.02 | 0.97 | 1.07 | 0.535 | 0.01 | 0.99 | 0.93 | 1.05 | 0.730 | 0.11 |
| CD8 naive abs | 1.00 | 1.00 | 1.01 | 0.226 | 0.05 | 1.00 | 1.00 | 1.01 | 0.659 | 0.11 |
| CD8+ T_EMRA_ (% of CD8+) | 0.98 | 0.94 | 1.03 | 0.493 | 0.01 | 1.00 | 0.95 | 1.05 | 0.878 | 0.11 |
| CD8+ T_EMRA_ abs | 1.00 | 1.00 | 1.00 | 0.932 | 0.00 | 1.00 | 1.00 | 1.00 | 0.767 | 0.11 |
| CD8+ T_EM_ (% of CD8+) | 0.97 | 0.88 | 1.06 | 0.532 | 0.01 | 0.98 | 0.89 | 1.07 | 0.623 | 0.11 |
| CD8+ T_EM_ abs | 1.00 | 0.99 | 1.01 | 0.998 | 0.00 | 1.00 | 0.99 | 1.01 | 0.906 | 0.11 |
| CD19 naive (% of B lymphocytes) | 0.99 | 0.94 | 1.03 | 0.548 | 0.01 | 1.00 | 0.95 | 1.05 | 0.931 | 0.11 |
| CD19 naive abs | 1.00 | 1.00 | 1.01 | 0.351 | 0.03 | 1.00 | 1.00 | 1.01 | 0.412 | 0.13 |
| CD19 non-switched memory (% of B lymphocytes) | 1.03 | 0.94 | 1.14 | 0.494 | 0.01 | 1.02 | 0.93 | 1.12 | 0.704 | 0.11 |
| CD19 non-switched memory abs | 1.02 | 1.00 | 1.04 | 0.095 | 0.09 | 1.01 | 0.99 | 1.04 | 0.184 | 0.16 |
| CD19 switched memory (% of B lymphocytes) | 1.03 | 0.95 | 1.13 | 0.425 | 0.02 | 1.01 | 0.92 | 1.11 | 0.818 | 0.11 |
| CD19 switched memory abs | 1.01 | 1.00 | 1.04 | 0.127 | 0.07 | 1.01 | 0.99 | 1.03 | 0.256 | 0.14 |
| CD19 memory CD27- (% of B lymphocytes) | 0.80 | 0.56 | 1.10 | 0.189 | 0.06 | 0.75 | 0.50 | 1.06 | 0.124 | 0.18 |
| CD19 memory CD27- abs | 1.00 | 0.92 | 1.09 | 0.915 | 0.00 | 0.99 | 0.90 | 1.08 | 0.847 | 0.11 |
| ***OLS regression models for MIDAS (values*** of ***0 to 100)*** | | | | | | | | | | |
| LEUKO | 0.98 | 0.83 | 1.14 | 0.752 | 0.00 | 0.96 | 0.83 | 1.11 | 0.570 | 0.17 |
| LYMPHO | 1.11 | 0.73 | 1.68 | 0.626 | 0.01 | 1.06 | 0.71 | 1.56 | 0.784 | 0.16 |
| Lymphocytes (% of leukocytes) | 1.01 | 1.00 | 1.03 | 0.106 | 0.06 | 1.01 | 0.99 | 1.03 | 0.189 | 0.20 |
| CD3 LEU (% of leukocytes) | 1.02 | 1.00 | 1.04 | 0.108 | 0.06 | 1.01 | 0.99 | 1.03 | 0.225 | 0.19 |
| CD3 abs | 1.24 | 0.95 | 1.62 | 0.118 | 0.06 | 1.16 | 0.89 | 1.51 | 0.261 | 0.19 |
| CD19 LEU (% of leukocytes) | 1.07 | 0.94 | 1.22 | 0.274 | 0.03 | 1.05 | 0.93 | 1.19 | 0.437 | 0.17 |
| CD19 abs | 2.41 | 0.43 | 13.35 | 0.307 | 0.02 | 1.67 | 0.32 | 8.67 | 0.534 | 0.17 |
| NK LEU (% of leukocytes) | 1.02 | 0.93 | 1.12 | 0.620 | 0.01 | 1.04 | 0.95 | 1.13 | 0.386 | 0.18 |
| NK abs | 1.30 | 0.36 | 4.61 | 0.683 | 0.00 | 1.43 | 0.43 | 4.77 | 0.548 | 0.17 |
| CD3 LYMFO (% of lymphocytes) | 1.02 | 0.98 | 1.06 | 0.257 | 0.03 | 1.01 | 0.97 | 1.04 | 0.690 | 0.16 |
| CD19 LYMFO (% of lymphocytes) | 0.96 | 0.88 | 1.04 | 0.317 | 0.02 | 0.97 | 0.89 | 1.04 | 0.377 | 0.18 |
| NK LYMFO (% of lymphocytes) | 0.99 | 0.95 | 1.02 | 0.456 | 0.01 | 1.00 | 0.96 | 1.04 | 0.992 | 0.16 |
| CD3+CD57+ (% of CD3+) | 0.98 | 0.96 | 1.01 | 0.256 | 0.03 | 0.99 | 0.96 | 1.02 | 0.660 | 0.16 |
| CD3+CD57+ (abs) | 0.84 | 0.28 | 2.54 | 0.755 | 0.00 | 1.14 | 0.38 | 3.36 | 0.812 | 0.16 |
| NK CD57+ (% of NK) | 0.99 | 0.98 | 1.01 | 0.461 | 0.01 | 1.00 | 0.98 | 1.02 | 0.994 | 0.16 |
| NK CD57+ abs | 0.57 | 0.07 | 4.81 | 0.597 | 0.01 | 0.87 | 0.11 | 6.72 | 0.893 | 0.16 |
| CD3+CD69+ (% of CD3+) | 1.03 | 0.93 | 1.14 | 0.578 | 0.01 | 1.03 | 0.94 | 1.14 | 0.493 | 0.17 |
| CD19+CD69+ (% of CD19+) | 1.00 | 0.82 | 1.22 | 0.985 | 0.00 | 0.96 | 0.80 | 1.16 | 0.673 | 0.16 |
| NK CD69+ (% of NK) | 1.00 | 0.97 | 1.03 | 0.932 | 0.00 | 1.00 | 0.97 | 1.02 | 0.730 | 0.16 |
| CD4 LEU (% of leukocytes) | 1.02 | 0.99 | 1.05 | 0.261 | 0.03 | 1.01 | 0.98 | 1.04 | 0.377 | 0.18 |
| CD4 abs | 1.26 | 0.86 | 1.85 | 0.224 | 0.03 | 1.18 | 0.82 | 1.70 | 0.357 | 0.18 |
| CD8 LEU (% of leukocytes) | 1.04 | 0.98 | 1.10 | 0.195 | 0.04 | 1.03 | 0.97 | 1.08 | 0.327 | 0.18 |
| CD8 abs | 1.70 | 0.77 | 3.73 | 0.183 | 0.04 | 1.43 | 0.66 | 3.11 | 0.356 | 0.18 |
| CD4 LYMFO (% of lymphocytes) | 1.00 | 0.97 | 1.03 | 0.881 | 0.00 | 1.00 | 0.97 | 1.03 | 0.851 | 0.16 |
| CD8 LYMFO (% of lymphocytes) | 1.02 | 0.98 | 1.05 | 0.284 | 0.03 | 1.01 | 0.98 | 1.05 | 0.380 | 0.18 |
| Treg (CD4) (% of CD4+) | 1.03 | 0.93 | 1.13 | 0.588 | 0.01 | 1.06 | 0.97 | 1.16 | 0.219 | 0.19 |
| Treg abs | 20.79 | 0.33 | 1292.20 | 0.146 | 0.05 | 28.85 | 0.61 | 1367.50 | 0.086 | 0.22 |
| Treg CD45RA+ (% of Treg) | 1.01 | 1.00 | 1.03 | 0.112 | 0.06 | 1.01 | 0.99 | 1.03 | 0.483 | 0.17 |
| CD4+ T_CM_ (% of CD4+) | 0.99 | 0.96 | 1.02 | 0.440 | 0.01 | 1.00 | 0.98 | 1.03 | 0.794 | 0.16 |
| CD4+ T_CM_ abs | 1.00 | 1.00 | 1.00 | 0.333 | 0.02 | 1.00 | 1.00 | 1.00 | 0.241 | 0.19 |
| CD4 naive (% of CD4+) | 1.01 | 0.99 | 1.03 | 0.447 | 0.01 | 1.00 | 0.97 | 1.02 | 0.677 | 0.16 |
| CD4 naive abs | 1.00 | 1.00 | 1.00 | 0.247 | 0.03 | 1.00 | 1.00 | 1.00 | 0.736 | 0.16 |
| CD4+ T_EMRA_ (% of CD4+) | 1.02 | 0.97 | 1.08 | 0.384 | 0.02 | 1.02 | 0.97 | 1.07 | 0.426 | 0.17 |
| CD4+ T_EMRA_ abs | 1.00 | 1.00 | 1.00 | 0.295 | 0.03 | 1.00 | 1.00 | 1.00 | 0.266 | 0.19 |
| CD4+ T_EM_ (% of CD4+) | 0.97 | 0.93 | 1.00 | 0.068 | 0.08 | 0.98 | 0.94 | 1.02 | 0.243 | 0.19 |
| CD4+ T_EM_ abs | 1.00 | 1.00 | 1.00 | 0.751 | 0.00 | 1.00 | 1.00 | 1.00 | 0.603 | 0.17 |
| CD8+ T_CM_ (% of CD8+) | 1.00 | 0.96 | 1.03 | 0.791 | 0.00 | 1.01 | 0.97 | 1.05 | 0.595 | 0.17 |
| CD8+ T_CM_ abs | 1.00 | 1.00 | 1.01 | 0.228 | 0.03 | 1.00 | 1.00 | 1.01 | 0.137 | 0.21 |
| CD8 naive (% of CD8+) | 1.00 | 0.98 | 1.02 | 0.904 | 0.00 | 0.98 | 0.96 | 1.01 | 0.162 | 0.20 |
| CD8 naive abs | 1.00 | 1.00 | 1.00 | 0.209 | 0.04 | 1.00 | 1.00 | 1.00 | 0.944 | 0.16 |
| CD8+ T_EMRA_ (% of CD8+) | 1.00 | 0.98 | 1.02 | 0.719 | 0.00 | 1.00 | 0.98 | 1.02 | 0.749 | 0.16 |
| CD8+ T_EMRA_ abs | 1.00 | 1.00 | 1.00 | 0.653 | 0.00 | 1.00 | 1.00 | 1.00 | 0.426 | 0.17 |
| CD8+ T_EM_ (% of CD8+) | 1.01 | 0.98 | 1.05 | 0.436 | 0.01 | 1.02 | 0.98 | 1.05 | 0.294 | 0.18 |
| CD8+ T_EM_ abs | 1.00 | 1.00 | 1.01 | 0.207 | 0.04 | 1.00 | 1.00 | 1.01 | 0.299 | 0.18 |
| CD19 naive (% of B lymphocytes) | 0.99 | 0.97 | 1.01 | 0.286 | 0.03 | 1.00 | 0.98 | 1.02 | 0.667 | 0.16 |
| CD19 naive abs | 1.00 | 1.00 | 1.00 | 0.653 | 0.00 | 1.00 | 1.00 | 1.00 | 0.773 | 0.16 |
| CD19 non-switched memory (% of B lymphocytes) | 1.02 | 0.99 | 1.06 | 0.223 | 0.04 | 1.01 | 0.98 | 1.05 | 0.422 | 0.17 |
| CD19 non-switched memory abs | 1.01 | 1.00 | 1.01 | 0.117 | 0.06 | 1.00 | 1.00 | 1.01 | 0.316 | 0.18 |
| CD19 switched memory (% of B lymphocytes) | 1.02 | 0.98 | 1.05 | 0.306 | 0.02 | 1.01 | 0.97 | 1.04 | 0.724 | 0.16 |
| CD19 switched memory abs | 1.01 | 1.00 | 1.01 | 0.132 | 0.05 | 1.00 | 1.00 | 1.01 | 0.352 | 0.18 |
| CD19 memory CD27- (% of B lymphocytes) | 0.95 | 0.83 | 1.08 | 0.391 | 0.02 | 0.93 | 0.82 | 1.05 | 0.233 | 0.19 |
| CD19 memory CD27- abs | 1.00 | 0.97 | 1.04 | 0.835 | 0.00 | 1.00 | 0.97 | 1.03 | 0.864 | 0.16 |
| ***Logistic regression models for HIT 6*** | | | | | | | | | | |
| LEUKO | 0.96 | 0.63 | 1.55 | 0.866 | 0.00 | 0.91 | 0.57 | 1.50 | 0.697 | 0.16 |
| LYMPHO | 1.46 | 0.45 | 5.82 | 0.556 | 0.01 | 1.24 | 0.38 | 4.61 | 0.729 | 0.16 |
| Lymphocytes (% of leukocytes) | 1.02 | 0.97 | 1.09 | 0.392 | 0.03 | 1.02 | 0.96 | 1.08 | 0.562 | 0.16 |
| CD3 LEU (% of leukocytes) | 1.04 | 0.97 | 1.11 | 0.305 | 0.04 | 1.02 | 0.96 | 1.10 | 0.499 | 0.17 |
| CD3 abs | 1.36 | 0.61 | 3.40 | 0.473 | 0.02 | 1.16 | 0.51 | 2.83 | 0.729 | 0.16 |
| CD19 LEU (% of leukocytes) | 1.08 | 0.75 | 1.65 | 0.700 | 0.01 | 1.03 | 0.70 | 1.57 | 0.874 | 0.15 |
| CD19 abs | 2.27 | 0.02 | 680.46 | 0.753 | 0.00 | 1.07 | 0.01 | 309.36 | 0.979 | 0.15 |
| NK LEU (% of leukocytes) | 0.96 | 0.74 | 1.26 | 0.746 | 0.00 | 0.99 | 0.75 | 1.34 | 0.962 | 0.15 |
| NK abs | 0.49 | 0.02 | 20.58 | 0.693 | 0.01 | 0.60 | 0.01 | 33.49 | 0.787 | 0.15 |
| CD3 LYMFO (% of lymphocytes) | 1.07 | 0.96 | 1.19 | 0.218 | 0.05 | 1.04 | 0.93 | 1.17 | 0.485 | 0.17 |
| CD19 LYMFO (% of lymphocytes) | 0.90 | 0.70 | 1.15 | 0.419 | 0.02 | 0.92 | 0.71 | 1.18 | 0.523 | 0.16 |
| NK LYMFO (% of lymphocytes) | 0.95 | 0.85 | 1.06 | 0.339 | 0.03 | 0.98 | 0.87 | 1.11 | 0.682 | 0.16 |
| CD3+CD57+ (% of CD3+) | 0.92 | 0.85 | 1.00 | 0.062 | 0.13 | 0.90 | 0.80 | 1.00 | 0.058 | 0.28 |
| CD3+CD57+ (abs) | 0.21 | 0.01 | 4.08 | 0.272 | 0.04 | 0.22 | 0.01 | 5.26 | 0.329 | 0.18 |
| NK CD57+ (% of NK) | 0.98 | 0.93 | 1.03 | 0.393 | 0.03 | 0.99 | 0.93 | 1.04 | 0.618 | 0.16 |
| NK CD57+ abs | 0.04 | 0.00 | 12.16 | 0.252 | 0.05 | 0.06 | 0.00 | 24.33 | 0.346 | 0.18 |
| CD3+CD69+ (% of CD3+) | 1.02 | 0.76 | 1.40 | 0.911 | 0.00 | 1.04 | 0.76 | 1.47 | 0.808 | 0.15 |
| CD19+CD69+ (% of CD19+) | 0.98 | 0.57 | 1.80 | 0.944 | 0.00 | 0.89 | 0.51 | 1.56 | 0.674 | 0.16 |
| NK CD69+ (% of NK) | 1.04 | 0.96 | 1.17 | 0.396 | 0.03 | 1.06 | 0.95 | 1.20 | 0.358 | 0.18 |
| CD4 LEU (% of leukocytes) | 1.06 | 0.97 | 1.20 | 0.249 | 0.05 | 1.05 | 0.96 | 1.18 | 0.330 | 0.19 |
| CD4 abs | 1.91 | 0.59 | 8.85 | 0.334 | 0.04 | 1.62 | 0.51 | 7.07 | 0.441 | 0.17 |
| CD8 LEU (% of leukocytes) | 1.11 | 0.93 | 1.38 | 0.321 | 0.04 | 1.05 | 0.88 | 1.32 | 0.620 | 0.16 |
| CD8 abs | 2.43 | 0.24 | 40.05 | 0.486 | 0.02 | 1.20 | 0.10 | 19.96 | 0.892 | 0.15 |
| CD4 LYMFO (% of lymphocytes) | 1.02 | 0.94 | 1.12 | 0.624 | 0.01 | 1.02 | 0.93 | 1.13 | 0.630 | 0.16 |
| CD8 LYMFO (% of lymphocytes) | 1.03 | 0.93 | 1.16 | 0.570 | 0.01 | 1.00 | 0.90 | 1.13 | 0.967 | 0.15 |
| Treg (CD4) (% of CD4+) | 0.76 | 0.55 | 1.00 | 0.065 | 0.13 | 0.74 | 0.50 | 1.01 | 0.086 | 0.26 |
| Treg abs | 1.36 | 0.00 | 4388958.85 | 0.960 | 0.00 | 2.45 | 0.00 | 2291290.70 | 0.880 | 0.15 |
| Treg CD45RA+ (% of Treg) | 1.05 | 0.99 | 1.13 | 0.104 | 0.11 | 1.04 | 0.97 | 1.12 | 0.290 | 0.19 |
| CD4+ T_CM_ (% of CD4+) | 0.99 | 0.91 | 1.07 | 0.738 | 0.00 | 1.04 | 0.94 | 1.14 | 0.466 | 0.17 |
| CD4+ T_CM_ abs | 1.00 | 1.00 | 1.01 | 0.346 | 0.04 | 1.00 | 1.00 | 1.01 | 0.310 | 0.20 |
| CD4 naive (% of CD4+) | 1.07 | 0.99 | 1.16 | 0.098 | 0.11 | 1.04 | 0.96 | 1.14 | 0.398 | 0.18 |
| CD4 naive abs | 1.00 | 1.00 | 1.00 | 0.248 | 0.05 | 1.00 | 1.00 | 1.00 | 0.534 | 0.16 |
| CD4+ T_EMRA_ (% of CD4+) | 1.10 | 0.93 | 1.43 | 0.366 | 0.04 | 1.09 | 0.91 | 1.41 | 0.443 | 0.17 |
| CD4+ T_EMRA_ abs | 1.01 | 1.00 | 1.03 | 0.198 | 0.09 | 1.01 | 1.00 | 1.03 | 0.207 | 0.22 |
| CD4+ T_EM_ (% of CD4+) | 0.82 | 0.69 | 0.93 | 0.010 | 0.33 | 0.84 | 0.70 | 0.95 | 0.019 | 0.38 |
| CD4+ T_EM_ abs | 1.00 | 0.99 | 1.01 | 0.801 | 0.00 | 1.00 | 0.99 | 1.01 | 0.980 | 0.15 |
| CD4+ T_CM_ (% of CD8+) | 1.05 | 0.94 | 1.22 | 0.417 | 0.03 | 1.15 | 1.00 | 1.40 | 0.084 | 0.27 |
| CD4+ T_CM_ abs | 1.01 | 1.00 | 1.04 | 0.148 | 0.09 | 1.01 | 1.00 | 1.04 | 0.138 | 0.24 |
| CD8 naive (% of CD8+) | 1.03 | 0.97 | 1.10 | 0.348 | 0.03 | 1.00 | 0.93 | 1.08 | 0.966 | 0.15 |
| CD8 naive abs | 1.00 | 1.00 | 1.01 | 0.466 | 0.02 | 1.00 | 0.99 | 1.01 | 0.787 | 0.15 |
| CD8+ T_EMRA_ (% of CD8+) | 0.96 | 0.91 | 1.02 | 0.160 | 0.07 | 0.96 | 0.90 | 1.02 | 0.211 | 0.20 |
| CD8+ T_EMRA_ abs | 1.00 | 1.00 | 1.01 | 0.953 | 0.00 | 1.00 | 1.00 | 1.01 | 0.945 | 0.15 |
| CD8+ T_EM_ (% of CD8+) | 1.02 | 0.92 | 1.15 | 0.716 | 0.00 | 1.04 | 0.93 | 1.17 | 0.550 | 0.16 |
| CD8+ T_EM_ abs | 1.00 | 0.99 | 1.02 | 0.453 | 0.02 | 1.00 | 0.99 | 1.02 | 0.656 | 0.16 |
| CD19 naive (% of B lymphocytes) | 0.98 | 0.92 | 1.04 | 0.558 | 0.01 | 1.00 | 0.93 | 1.06 | 0.944 | 0.15 |
| CD19 naive abs | 1.00 | 0.99 | 1.01 | 0.982 | 0.00 | 1.00 | 0.99 | 1.01 | 0.954 | 0.15 |
| CD19 non-switched memory (% of B lymphocytes) | 1.03 | 0.93 | 1.17 | 0.571 | 0.01 | 1.01 | 0.90 | 1.14 | 0.913 | 0.15 |
| CD19 non-switched memory abs | 1.01 | 0.99 | 1.04 | 0.409 | 0.03 | 1.00 | 0.98 | 1.04 | 0.749 | 0.15 |
| CD19 switched memory (% of B lymphocytes) | 1.01 | 0.92 | 1.14 | 0.785 | 0.00 | 0.99 | 0.88 | 1.12 | 0.813 | 0.15 |
| CD19 switched memory abs | 1.00 | 0.98 | 1.03 | 0.862 | 0.00 | 1.00 | 0.97 | 1.02 | 0.785 | 0.15 |
| CD19 memory CD27- (% of B lymphocytes) | 1.22 | 0.83 | 1.90 | 0.336 | 0.03 | 1.19 | 0.80 | 1.88 | 0.412 | 0.17 |
| CD19 memory CD27- abs | 1.05 | 0.95 | 1.20 | 0.392 | 0.03 | 1.04 | 0.94 | 1.18 | 0.510 | 0.17 |

Legend: BETA is the estimated regression coefficient. For logistic regression models, the odds ratio (exponentiated regression coefficient) is reported. 95% LCI/UCI BETA are the 95% lower and upper confidence interval estimates. R2 is the coefficient of determination for the OLS model and the pseudo coefficient of determination for the logistic models.

**Supplementary figures**

**Gating strategy for immune cell subsets**
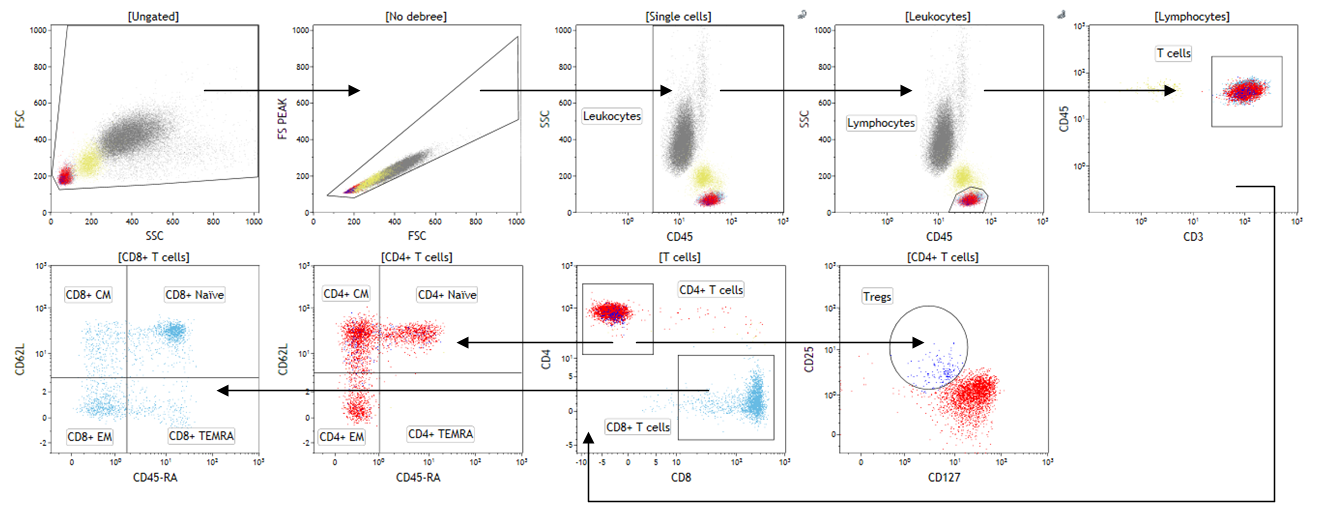


**Suppl. Figure 1. Gating of subpopulations of T cells:** Obtained events were gated in FSC intensity and SSC intensity dot plot to eliminate debris. Cells were gated on FSC intensity and FSC peak dot plot to eliminate doublets. Leukocytes were gated on SSC vs. CD45 dot plot to eliminate events of non-leukocyte origin. Lymphocytes were gated on SSC vs. CD45 dot plot as SSC low / CD45high cells. T cells were gated as CD3+ cells within lymphocytes. CD4+ and CD8+ cells were gated within T cells. Subsequently, CD4+ and CD8+ T cells were divided into subpopulations according to the expression of CD62L and CD45RA in this manner: naive (CD45RA+/CD62L+), terminally differentiated (CD45RA+/CD62L-), central memory (CD45RA-/CD62L+) and effector memory T cells (CD45RA-/CD62L-). Tregs were gated as CD4+/CD25+/CD127- T cells.

FSC: Forward scatter, SSC: Side scatter.


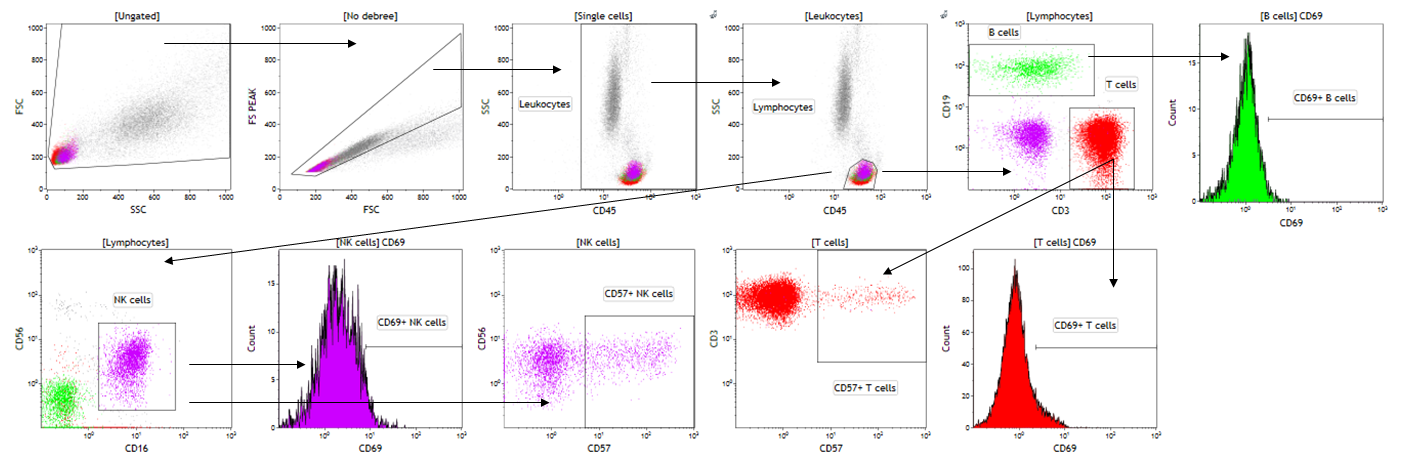


**Suppl. Figure 2. Gating of subpopulations of lymphocytes and evaluating expression of markers of activation and exhaustion:** Obtained events were gated in FSC intensity and SSC intensity dot plot to eliminate debris. Cells were gated on FSC intensity and FSC peak dot plot to eliminate doublets. Leukocytes were gated on SSC vs. CD45 dot plot to eliminate events of non-leukocyte origin. Lymphocytes were gated on SSC vs. CD45 dot plot as SSC low / CD45high cells. T cells were gated as CD3+ cells within lymphocytes. B cells were gated as CD19+ lymphocytes. NK cells were gated as CD16+/CD56+ lymphocytes. Then expression of CD57 was evaluated on surface of T cells and NK cells and expression of CD69 was evaluated on surface of T cells, B cells and NK cells (shown as histograms).

FSC: Forward scatter, SSC: Side scatter.


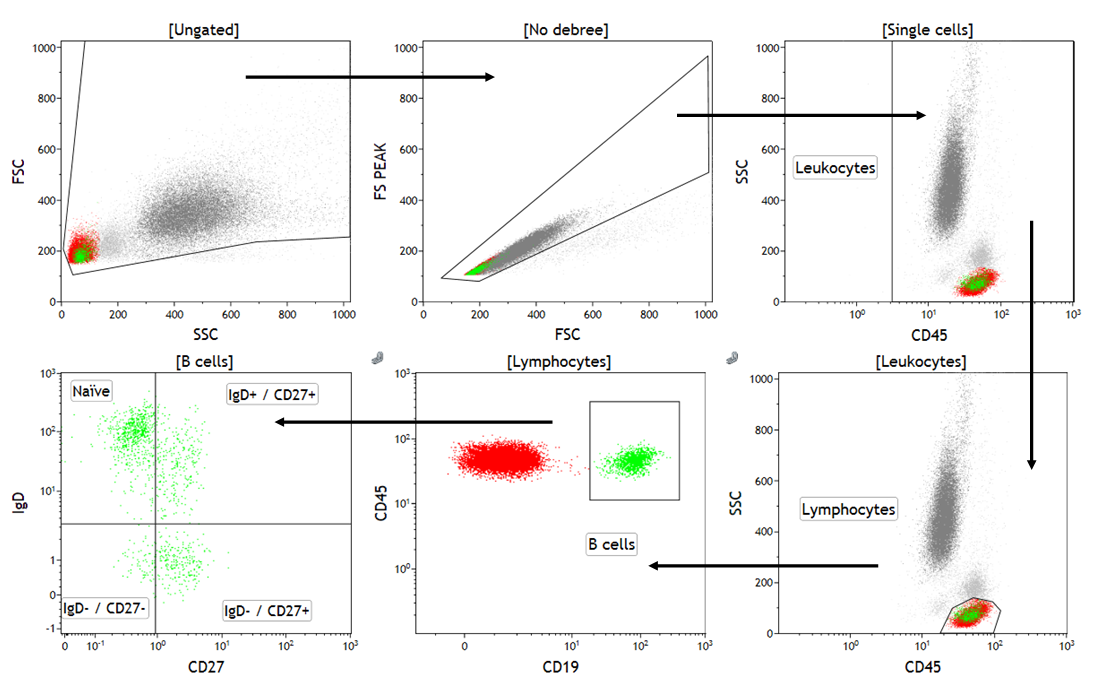


**Suppl. Figure 3. Gating of subpopulations of B cells:** Obtained events were gated in FSC intensity and SSC intensity dot plot to eliminate debris. Cells were gated on FSC intensity and FSC peak dot plot to eliminate doublets. Leukocytes were gated on SSC vs. CD45 plot to eliminate events of non-leukocyte origin. Lymphocytes were gated on SSC vs. CD45 dot plot as SSC low / CD45high cells. B cells were gated as CD19 cells within lymphocytes. Based on IgD and CD27 expression, B cells were divided into these subtypes: naive B cells (IgD+/CD27+), non-switched memory B cells (IgD+/CD27+), class-switched memory B cells (IgD-/CD27+) and double-negative B cells (IgD-/CD27-).

FSC: Forward scatter, SSC: Side scatter.

**Suppl. Figure 4.** Boxplot of 4 parameters correlated with HIT6

**
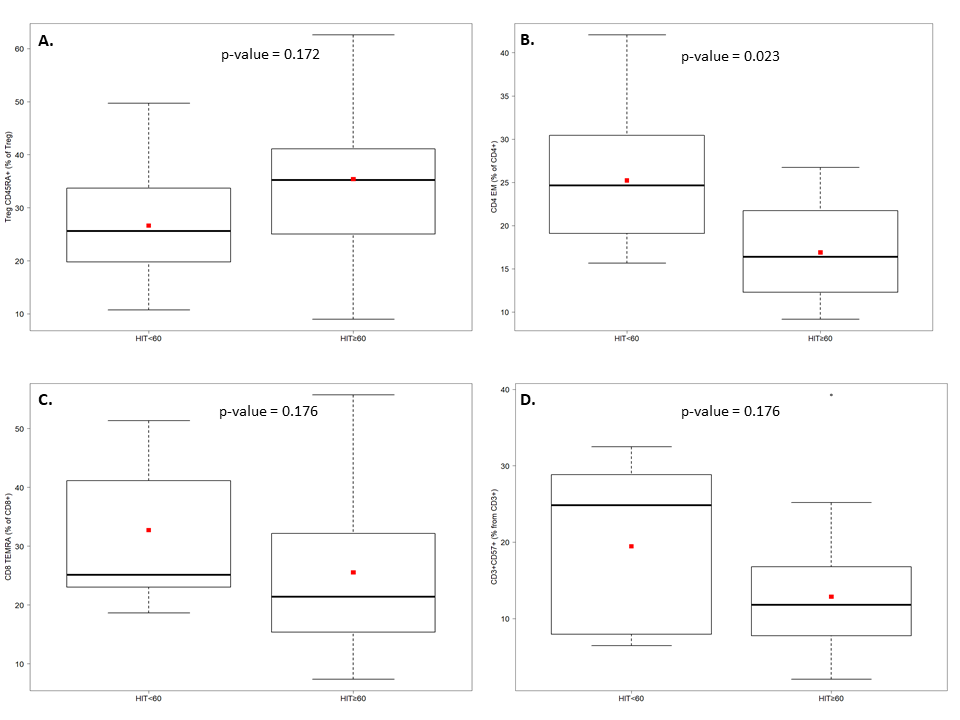
**

Legend: Horizontal lines in boxplots shows quantiles (bold line is median) and red box mean for patients with HIT<60 and HIT≥60 separately. Figure **A** is boxplot for Treg CD45RA+ (% of Treg), **B** for CD4 EM (% of CD4+), **C** for CD8 TEMRA (% of CD8+), **D** for CD3+CD57+ (% of CD3+). All P values (two-sided alternative hypothesis, statistically tested by Mann-Whitney U test) are reported after Benjamini-Hochberg correction.
